# Association of Changes in Metabolic Syndrome Status With the Incidence of Thyroid Nodules: A Prospective Study in Chinese Adults

**DOI:** 10.3389/fendo.2020.00582

**Published:** 2020-08-21

**Authors:** Qijun Liang, Shouyi Yu, Shihui Chen, Yan Yang, Shuhua Li, Chenming Hu, Danxuan Huang, Li Kuang, Dongcai Li

**Affiliations:** Health Management Center, Foshan Hospital of Traditional Chinese Medicine, Foshan, China

**Keywords:** metabolic syndrome, thyroid nodules, gender, age, prospective study

## Abstract

Although several cross-sectional studies have shown an association of metabolic syndrome (MetS) with nodular thyroid disease, related prospective studies are scarce. This study investigated the association of MetS with thyroid nodule (TN) incidence in Chinese adults, and explored whether the development of or recovery from MetS is associated with changes in the risk of developing TNs. A total of 4,749 Chinese aged 18–65 years were involved in this 6-year prospective study. The association of MetS with TN prevalence was examined. TN-free individuals at baseline (*n* = 3,133) were further examined. TN incidence rates in groups with different MetS statuses (MetS-free, MetS-developed, MetS-recovery and MetS-chronic) were analyzed. Of all participants, 18.21 and 31.65% had MetS and TNs, respectively. MetS patients had a higher TN prevalence than the non-MetS group (31.08 vs. 19.81% in males, *p* < 0.01; 59.52 vs. 39.59% in females, *p* < 0.01). Sex, age and MetS were independent risk factors for TNs. At a median follow up of 5.94 years, the MetS-chronic group (4.37/100 person-years) had a higher risk of TNs (adjusted incidence rate ratio [IRR] = 1.288 [95% CI 1.014–1.636]) compared with the MetS-free group (2.72/100 person-years) in the whole cohort. In males, the MetS-chronic group (3.76/100 person-years) had a higher risk of TNs (adjusted IRR = 1.367 [95% CI 1.017–1.835]) compared with the MetS-free group (2.31/100 person-years). In females, the risk of TNs was significantly higher in the MetS-chronic (6.44/100 person-years) and MetS-developed (6.31/100 person-years) groups compared with the MetS-free group (3.23/100 person-years).

## Introduction

In the past 30 years, thyroid nodules (TNs) with an increasing incidence have become a common thyroid disorder detected by ultrasound in 20–67% of individuals (1). Advances in diagnostic tools, including high-resolution ultrasonography and computed tomography, may partly explain this increasing trend (2). However, changes in the intrinsic characteristics of the general population are likely to be also involved (3).

Similarly, the incidence of metabolic syndrome (MetS), which is characterized by a cluster of metabolic risk factors (e.g., central obesity, hypertension, hyperglycemia, and dyslipidemia), has been rising exponentially in the last decades due to economic development and changes in human diets and lifestyles (4). Since the insulin/insulin-like growth factor axis simulates the proliferation of thyroid follicular cells (5, 6), it was hypothesized that compensatory hyperinsulinemia following insulin resistance in previous MetS might be responsible for the rising trend of TNs (7, 8). In addition, increased incidence in TNs could be due to growing exposure to certain toxicants that may act as thyroid disruptors (9), and age may be another risk factor for TN development (10, 11).

Although several reports have demonstrated an association of MetS with TNs, they were mainly cross-sectional studies designed to evaluate either the prevalence of MetS in individuals with TNs vs. controls (12–15), or, conversely, the prevalence of TNs in MetS patients (16, 17). Meanwhile, related prospective studies are scarce.

Therefore, this 6-year prospective study aimed to investigate the association of MetS with the incidence of TNs in Chinese adults. Since the MetS status is known to change dynamically (18–20), we also explored whether the development of or recovery from MetS is associated with an altered risk for TNs.

## Methods

### Study Population

This population-based prospective cohort study was initiated in January 1st, 2013. Chinese adults aged 18–65 years undergoing a routine medical health check-up at the Health Management Center of Foshan Hospital of Traditional Chinese Medicine were enrolled. Exclusion criteria were: (a) a history of other thyroid diseases, including hyperthyroidism, hypothyroidism, subacute thyroiditis, and Hashimoto thyroiditis; (b) a history of thyroid therapy, including medicines, operation, or radiotherapy for head and neck disease; (c) a history of other endocrine diseases, glucocorticoid treatment, or hormone replacement therapy; (d) chronic diseases (cardiac failure, hepatic, or renal dysfunction), or significant mental or neurological disorders; (e) a history of cancer; (f) pregnancy or lactating in women; (g) exposure to iodinated contrast material in the previous 6 months; (h) a history of taking amiodarone, smoking over 3 months, or drinking in the last 6 months (alcohol intake >25 g/day for men and >15 g/day for women). The study protocol was approved by the Committee on Human Research at Foshan Hospital of Traditional Chinese Medicine. Written informed consent was obtained from each participant.

### Definition and Diagnostic Criteria

MetS was defined according to the updated National Cholesterol Education Program Panel III criteria for Asian-Americans (21). Thyroid nodules were detected as previously described, with diameters equal to or exceeding 2 mm (22). Non-alcoholic fatty liver disease (NAFLD) was defined according to the “Diagnostic Criteria of Non-alcoholic Fatty Liver Disease by the Chinese Society of Hepatology” after exclusion of viral or autoimmune liver disease and excessive alcohol consumption (23), based on hepatic ultrasonography (24).

### Study Groups

In the prospective part of this study, the participants were divided into 4 groups according to MetS statuses at the beginning of and after a 6-year follow up: MetS-free (individuals consistently without MetS during follow up), MetS-developed (individuals with newly developed MetS), MetS-recovery (individuals recovering from preexisting MetS), and MetS-chronic (individuals with MetS throughout the study).

### Data Collection

Data on demography, health status, and lifestyle were collected using a standard questionnaire. Body weight and height were measured with the participant dressed in light clothing without shoes. Body mass index was determined as weight divided by height squared (kg/m^2^). Waist circumference (WC) was measured at the midpoint between the lower edge of the costal arch and the top of the iliac crest in the standing position. After 10 min rest, blood pressure was measured thrice at the right arm, and averaged.

### Laboratory Measurements

Blood samples were collected after overnight fasting at baseline and at the end of the follow-up. Clinical biomarkers, including fasting plasma glucose (FPG), total cholesterol (TC), triglycerides (TG), low density lipoprotein cholesterol (LDL-C), and high density lipoprotein cholesterol (HDL-C) were measured on an automatic analyzer (Hitachi, Tokyo, Japan). Specifically, FPG was measured by the hexokinase/G-6-PDH method, and TG and TC by the peroxidase-chromogen method using kinase-glycerol-3-phosphate and cholesterol esterase–cholesterol oxidase, respectively. LDL-C and HDL-C were measured by the direct one-step method.

### Thyroid Ultrasonography

In the supine position with the hyperextended neck on a pillow, ultrasound examination of TNs was performed, assessing TN number and location, by senior experts on a B-mode high-resolution tomographic ultrasound system (Esaote, Genova, Italy).

### Follow Up

The participants were followed up once a year, with text messages reminding them to return to the hospital for medical examination and providing lifestyle guidance. Individuals with abnormal metabolism were also treated during the follow up visits. The follow-up period was 6 years.

### Statistical Analysis

Continuous variables are mean ± standard deviation (SD), and categorical variables were presented as number (percentage). *T*-test, ANOVA and Pearson's chi-square test were performed to analyze differences in means and proportions between or between groups as appropriate. Multivariate binary logistic regression analysis was performed to assess risk factors for TN prevalence at baseline. Poisson regression was applied to calculate incidence rate ratios (IRRs) and 95% confidence intervals (CIs). All statistical analyses were carried out with SPSS 25 (SPSS, USA). Two-sided *P* < 0.05 was considered statistically significant.

## Results

### General Characteristics of all Subjects and Risk Factors for TNs

A total of 4,749 subjects were included at baseline. There were 2,525 males and 2,224 females, aged 37.54 ± 10.07 years. These participants were stratified by gender and MetS status (Table 1) and further analyzed. In all subjects, the prevalence of MetS was 18.21%. MetS prevalence was significantly higher in males compared with females (22.81 vs. 12.99%, *p* < 0.01).

**Table 1:** Clinical characteristics of subjects according to the MetS status stratified by gender.

**Parameters**	**Total subjects (*****n*** **=** **4749)**		***P***	**Males (*****n*** **=** **2525)**		***P***	**Females (*****n*** **=** **2224)**		***P***
	**MetS(–)**	**MetS(+)**		**MetS(–)**	**MetS(+)**		**MetS(–)**	**MetS(+)**	
*n*	3,884	865		1,949	576		1,935	289	
Age (years)	36.61 ± 9.98	41.72 ± 9.44	<0.001	37.02 ± 9.90	40.02 ± 8.97	<0.001	36.20 ± 10.04	45.11 ± 9.47	<0.001
Height (cm)	163.37 ± 8.14	165.45 ± 8.46	0.001	168.94 ± 6.48	169.65 ± 6.22	0.02	157.76 ± 5.30	157.09 ± 5.67	0.048
Weight (kg)	60.54 ± 10.74	73.30 ± 11.18	<0.001	67.00 ± 9.62	78.00 ± 9.40	<0.001	54.02 ± 7.33	63.93 ± 8.16	<0.001
BMI (kg/cm2)	22.59 ± 2.99	26.67 ± 2.70	<0.001	23.46 ± 2.96	27.07 ± 2.54	<0.001	21.71 ± 2.76	25.89 ± 2.84	<0.001
WC (cm)	81.05 ± 6.69	89.38 ± 6.41	<0.001	85.17 ± 5.85	92.20 ± 5.06	<0.001	76.90 ± 4.60	83.77 ± 4.96	<0.001
SBP (mmHg)	117.79 ± 12.69	134.13 ± 15.00	<0.001	121.30 ± 11.98	133.93 ± 13.37	<0.001	114.26 ± 12.40	134.53 ± 17.82	<0.001
DBP (mmHg)	70.79 ± 8.57	81.22 ± 10.28	<0.001	72.83 ± 8.55	82.06 ± 9.95	<0.001	68.74 ± 8.08	79.55 ± 10.73	<0.001
FPG (mmol/L)	5.31 ± 0.63	6.15 ± 1.68	<0.001	5.39 ± 0.74	6.20 ± 1.74	<0.001	5.23 ± 0.49	6.05 ± 1.55	0.508
TG (mmol/L)	1.16 ± 0.90	2.70 ± 2.71	<0.001	1.41 ± 1.09	2.96 ± 3.17	<0.001	0.90 ± 0.55	2.18 ± 1.26	<0.001
TCH (mmol/L)	4.90 ± 0.93	5.38 ± 1.09	<0.001	4.97 ± 0.91	5.43 ± 1.14	<0.001	4.83 ± 0.94	5.29 ± 0.99	0.01
LDL-C (mmol/L)	2.84 ± 0.80	3.17 ± 0.88	<0.001	2.98 ± 0.78	3.18 ± 0.89	<0.001	2.71 ± 0.80	3.15 ± 0.86	0.002
HDL-C (mmol/L)	1.47 ± 0.24	1.26 ± 0.20	<0.001	1.38 ± 0.21	1.24 ± 0.19	<0.001	1.55 ± 0.24	1.29 ± 0.20	<0.001
UA (μmol/L)	346.69 ± 93.05	411.96 ± 100.42	<0.001	401.68 ± 84.11	444.00 ± 97.03	<0.001	291.30 ± 64.35	348.10 ± 72.85	<0.001
NAFLD, *n* (%)	531 (13.67)	520 (60.12)	<0.001	409 (20.99)	387 (67.19)	<0.001	122 (6.30)	133 (46.02)	<0.001
TNs, *n* (%)	1,152 (29.66)	351 (40.58)	<0.001	386 (19.81)	179 (31.08)	<0.001	766 (39.59)	172 (59.52)	<0.001

*BMI, body mass index; WC, waist circumference; SBP, systolic blood pressure; DBP, diastolic blood pressure; FPG, fasting plasma glucose; TG, triglyceride; TCH, total cholesterol; LDL-C, Low-density lipoprotein cholesterol; HDL-C, high-density lipoprotein cholesterol; UA, uric acid; NAFLD, nonalcoholic fatty liver disease; TNs, thyroid nodules; MetS, metabolic syndrome*.

Based on thyroid ultrasonography, the prevalence of TNs was 31.65% in the whole cohort, and significantly higher in females than in males (42.18 vs. 22.38%, *p* < 0.01). In addition, the prevalence of TNs was significantly increased in the MetS group compared with MetS-free individuals (40.58 vs. 29.66% in all subjects, *p* < 0.01; 31.08 vs. 19.81% in males, *p* < 0.01; 59.52 vs. 39.59% in females, *p* < 0.01). Multivariate logistic regression analysis revealed that sex, age and MetS were independent risk factors for TNs in the whole cohort (Table 2). After stratified analysis by gender, age, and MetS were still independently associated with TN prevalence in both males and females.

**Table 2:** Multivariate analysis of potential risk factors for TNs in the whole study cohort.

	**OR (95% CI)**	***P***
**TOTAL SUBJECTS**		
Sex	2.883 (2.522–3.294)	<0.001
Age	1.056 (1.049–1.063)	<0.001
MetS	1.550 (1.310–1.832)	<0.001
**MALES**		
Age	1.041 (1.031–1.051)	<0.001
MetS	1.656 (1.339–2.048)	<0.001
**FEMALES**		
Age	1.070 (1.060–1.080)	<0.001
MetS	1.326 (1.010–1.739)	0.042

*MetS, metabolic syndrome; OR, odds ratio; CI, confidence interval*.

### Characteristics of the Followed Up Participants

After thyroid ultrasound, the TN-free subjects were enrolled in a follow up prospective study that ended on December 31^st^, 2019. There were 3,246 participants without TNs at baseline. During the study process, 13 women became pregnant, six individuals had Graves' disease, 27 participants had Hashimoto thyroiditis, one individual had subacute thyroiditis, and 66 subjects quit for personal reasons. Finally, 3,133 participants (1,900 males and 1,233 females) were followed up with a median time of 5.94 years (interquartile range of 5.84–6.05 years).

The baseline (2013) and follow up (2019) data of the 3,133 participants according to MetS status are shown in Table 3. In all participants, the MetS-free group included the highest percentage of females and the youngest population, while the MetS-chronic group included the elderly population. In male participants, the MetS-free group consisted of youngest individuals, while the MetS-recovery and MetS-chronic groups included the elderly population. In female participants, the MetS-free group included the youngest population, and the MetS-chronic group consisted of elderly individuals.

**Table 3:** Baseline and follow up characteristics of the participants according to the MetS status, stratified by gender.

**Parameters**	**Year**	**Total participants (*****n*** **=** **3,133)**				***P***	**Males (*****n*** **=** **1,900)**				***P***	**Females (*****n*** **=** **1,233)**				***P***
		**MetS-free**	**MetS-developed**	**MetS-recovery**	**MetS-chronic**		**MetS-free**	**MetS-developed**	**MetS-recovery**	**MetS-chronic**		**MetS-free**	**MetS-developed**	**MetS-recovery**	**MetS-chronic**	
*N*		2,368	261	194	310		1,306	205	149	240		1,062	56	45	70	
Male, *n* (%)		1,306 (55.15)	205 (78.54)	149 (76.80)	240 (77.42)	<0.001										
Age (years)	2013	34.71 ± 9.41	38.36 ± 10.65	39.91 ± 9.45	40.47 ± 9.47	<0.001	35.90 ± 9.66	37.87 ± 10.71	39.93 ± 9.47	39.10 ± 8.87	<0.001	33.24 ± 8.87	40.13 ± 10.31	39.85 ± 9.47	45.17 ± 10.00	<0.001
Height (cm)	2013	164.14 ± 7.94	167.31 ± 7.85	167.02 ± 7.80	167.63 ± 7.98	<0.001	168.99 ± 6.36	169.72 ± 6.57	169.61 ± 6.56	170.45 ± 6.06	0.007	158.17 ± 5.12	158.47 ± 5.48	158.44 ± 4.94	157.97 ± 5.96	0.938
	2019	164.31 ± 7.94	167.56 ± 7.81	167.26 ± 7.76	167.90 ± 7.85	<0.001	169.12 ± 6.40	169.99 ± 5.50	169.76 ± 6.67	170.62 ± 6.10	0.004	158.38 ± 5.16	158.70 ± 5.44	158.98 ± 4.83	158.56 ± 5.72	0.854
Weight (kg)	2013	60.14 ± 10.30	71.48 ± 10.82	72.65 ± 9.99	76.67 ± 10.61	<0.001	65.76 ± 9.06	73.91 ± 10.12	75.67 ± 8.66	79.99 ± 8.97	<0.001	53.23 ± 6.99	62.58 ± 8.40	62.64 ± 7.21	65.31 ± 7.53	<0.001
	2019	61.18 ± 10.35	74.17 ± 10.46	71.12 ± 10.44	77.22 ± 11.20	<0.001	66.78 ± 9.14	76.45 ± 9.42	74.13 ± 8.96	80.78 ± 9.44	<0.001	54.30 ± 7.10	65.81 ± 9.88	61.16 ± 8.69	65.00 ± 7.59	<0.001
BMI (kg/cm^2^)	2013	22.23 ± 2.84	25.46 ± 2.92	25.97 ± 2.52	27.20 ± 2.56	<0.001	23.00 ± 2.76	25.63 ± 3.02	26.28 ± 2.35	27.50 ± 2.31	<0.001	21.28 ± 2.65	24.84 ± 2.41	24.96 ± 2.77	26.21 ± 3.08	<0.001
	2019	22.57 ± 2.82	26.34 ± 2.66	25.35 ± 2.71	27.29 ± 2.68	<0.001	23.33 ± 2.75	26.42 ± 2.62	25.70 ± 2.47	27.71 ± 2.48	<0.001	21.64 ± 2.63	26.03 ± 2.81	24.18 ± 3.15	25.87 ± 2.88	<0.001
WC (cm)	2013	80.71 ± 6.45	87.93 ± 7.29	89.19 ± 5.81	91.82 ± 6.31	<0.001	84.27 ± 5.49	89.32 ± 7.20	90.97 ± 4.81	93.56 ± 5.48	<0.001	76.33 ± 4.59	82.85 ± 5.08	83.32 ± 4.91	85.85 ± 5.25	<0.001
	2019	81.28 ± 6.47	89.65 ± 6.74	88.26 ± 5.98	92.17 ± 6.43	<0.001	84.86 ± 5.45	91.06 ± 6.58	90.05 ± 4.92	93.98 ± 5.56	<0.001	76.88 ± 4.65	84.49 ± 4.40	82.37 ± 5.41	85.97 ± 5.28	<0.001
SBP (mmHg)	2013	116.70 ± 11.72	123.08 ± 11.08	130.59 ± 13.28	134.38 ± 14.09	<0.001	120.59 ± 10.92	124.55 ± 10.92	132.52 ± 11.33	134.30 ± 13.52	<0.001	111.92 ± 10.87	117.71 ± 10.00	124.18 ± 16.94	135.09 ± 16.00	<0.001
	2019	118.71 ± 12.87	132.01 ± 12.34	127.56 ± 14.15	136.40 ± 14.81	<0.001	122.60 ± 12.22	132.59 ± 11.85	129.62 ± 13.55	136.72 ± 13.33	<0.001	113.93 ± 12.00	129.89 ± 13.89	120.71 ± 14.07	135.33 ± 19.10	<0.001
DBP (mmHg)	2013	70.21 ± 8.20	74.96 ± 7.86	79.79 ± 9.39	82.02 ± 10.38	<0.001	72.28 ± 8.08	75.73 ± 7.90	80.97 ± 8.60	82.60 ± 10.43	<0.001	67.67 ± 7.63	72.14 ± 7.08	75.89 ± 10.88	80.05 ± 10.05	<0.001
	2019	69.94 ± 8.82	77.31 ± 9.58	76.49 ± 10.43	81.96 ± 11.33	<0.001	72.01 ± 8.84	78.03 ± 9.77	78.12 ± 10.26	82.76 ± 11.21	<0.001	67.38 ± 8.10	74.68 ± 8.42	71.09 ± 9.17	79.23 ± 11.41	<0.001
FPG(mmol/L)	2013	5.32 ± 0.48	5.65 ± 1.27	5.97 ± 1.00	6.24 ± 1.55	<0.001	5.38 ± 0.51	5.69 ± 1.40	6.06 ± 1.10	6.13 ± 1.11	<0.001	5.24 ± 0.43	5.51 ± 0.55	5.77 ± 0.45	6.64 ± 2.49	<0.001
	2019	4.99 ± 0.60	5.49 ± 1.38	5.37 ± 1.27	6.23 ± 2.32	<0.001	5.07 ± 0.65	5.50 ± 1.50	5.44 ± 1.43	6.24 ± 2.45	<0.001	4.90 ± 0.52	5.42 ± 0.74	5.15 ± 0.39	6.22 ± 1.80	<0.001
TG(mmol/L)	2013	1.07 ± 0.81	1.95 ± 1.43	2.37 ± 1.39	2.90 ± 1.84	<0.001	1.28 ± 0.95	2.12 ± 1.52	2.41 ± 1.48	3.11 ± 1.92	<0.001	0.81 ± 0.49	1.34 ± 0.72	2.27 ± 1.06	2.19 ± 1.33	<0.001
	2019	1.16 ± 0.71	2.57 ± 1.83	1.59 ± 0.81	2.91 ± 2.31	<0.001	1.34 ± 0.83	2.70 ± 1.97	1.65 ± 0.86	3.16 ± 2.43	<0.001	0.95 ± 0.45	2.07 ± 1.02	1.39 ± 0.59	2.09 ± 1.57	<0.001
TCH(mmol/L)	2013	4.82 ± 0.89	4.96 ± 0.84	5.40 ± 0.94	5.38 ± 1.09	<0.001	4.93 ± 0.92	4.95 ± 0.84	5.51 ± 0.94	5.41 ± 1.07	<0.001	4.68 ± 0.83	4.98 ± 0.87	5.04 ± 0.87	5.30 ± 1.18	<0.001
	2019	4.94 ± 0.88	5.07 ± 0.89	5.22 ± 0.96	5.38 ± 1.09	<0.001	5.06 ± 0.89	5.01 ± 0.86	5.27 ± 0.99	5.40 ± 1.07	<0.001	4.81 ± 0.84	5.29 ± 0.97	5.06 ± 0.86	5.33 ± 1.15	<0.001
LDL-C(mmol/L)	2013	2.76 ± 0.76	2.88 ± 0.76	3.18 ± 0.84	3.11 ± 0.92	<0.001	2.93 ± 0.78	2.90 ± 0.77	3.28 ± 0.82	3.11 ± 0.93	<0.001	2.55 ± 0.68	2.83 ± 0.74	2.86 ± 0.83	3.12 ± 0.92	<0.001
	2019	2.9 ± 0.77	3.05 ± 0.81	3.22 ± 0.84	3.18 ± 0.96	<0.001	3.08 ± 0.78	3.03 ± 0.82	3.30 ± 0.86	3.16 ± 0.95	0.005	2.68 ± 0.70	3.12 ± 0.78	2.96 ± 0.73	3.24 ± 0.97	<0.001
HDL-C(mmol/L)	2013	1.49 ± 0.22	1.33 ± 0.16	1.33 ± 0.19	1.29 ± 0.16	<0.001	1.42 ± 0.20	1.28 ± 0.13	1.32 ± 0.17	1.27 ± 0.14	<0.001	1.58 ± 0.22	1.50 ± 0.17	1.36 ± 0.24	1.36 ± 0.19	<0.001
	2019	1.39 ± 0.31	1.06 ± 0.22	1.24 ± 0.23	1.08 ± 0.22	<0.001	1.28 ± 0.27	1.00 ± 0.18	1.21 ± 0.21	1.05 ± 0.20	<0.001	1.52 ± 0.30	1.26 ± 0.24	1.32 ± 0.27	1.21 ± 0.23	<0.001
UA(μmol/L)	2013	346.11 ± 90.66	402.21 ± 100.51	406.25 ± 103.39	431.67 ± 98.90	<0.001	396.43 ± 79.62	423.03 ± 96.61	428.08 ± 101.49	457.60 ± 90.98	<0.001	284.22 ± 59.90	325.99 ± 74.90	333.98 ± 72.69	342.79 ± 69.12	<0.001
	2019	362.48 ± 93.81	433.83 ± 84.94	402.77 ± 97.54	447.60 ± 96.58	<0.001	413.31 ± 81.86	451.54 ± 78.13	422.01 ± 92.40	471.90 ± 86.07	<0.001	299.97 ± 65.57	369.01 ± 77.57	339.09 ± 87.24	364.27 ± 83.74	<0.001
NAFLD, n(%)	2013	254 (10.73)	98 (37.55)	106 (54.64)	210 (67.74)	<0.001	219 (16.77)	86 (41.95)	93 (62.42)	173 (72.08)	<0.001	35 (3.30)	12 (21.43)	13 (28.89)	37 (52.86)	<0.001
	2019	375 (15.84)	163 (62.45)	85 (43.81)	240 (77.42)	<0.001	321 (24.58)	129 (62.93)	72 (48.32)	199 (82.92)	<0.001	54 (5.08)	34 (60.71)	13 (28.89)	41 (58.57)	<0.001

*BMI, body mass index; WC, waist circumference; SBP, systolic blood pressure; DBP, diastolic blood pressure; FPG, fasting plasma glucose; TG, triglyceride; TCH, total cholesterol; LDL-C, Low-density lipoprotein cholesterol; HDL-C, high-density lipoprotein cholesterol; UA, uric acid; NAFLD, nonalcoholic fatty liver disease; MetS, metabolic syndrome*.

### Risk of TNs According to the Dynamic MetS Status

In all participants, TN incidence was significantly higher in the MetS-chronic group (4.37/100 person-years) compared with the MetS-free group (2.72/100 person-years) throughout follow up. The MetS-free, MetS-developed, and MetS-recovery groups were similar in TN incidence. Similar results were obtained after adjustment for age and sex (Table 4).

**Table 4:** Risk of TNs according to the dynamic MetS status.

**MetS status**	**Event, *n***	**Person-years**	**Incidence rate per 100 person-years**	**Unadjusted model**		Multivariable model^*^	
				**IRR (95% CI)**	***P***	**Adjusted IRR (95% CI)**	***P***
**TOTAL PARTICIPANTS**							
MetS-free	383	14,099	2.72	1 (reference)		1 (reference)	
MetS-developed	55	1,567	3.51	1.187 (0.899–1.568)	0.227	1.142 (0.863–1.513)	0.353
MetS-recovery	42	1,158	3.63	1.224 (0.894–1.677)	0.207	1.094 (0.797–1.501)	0.579
MetS-chronic	81	1,855	4.37	1.530 (1.209–1.937)	<0.001	1.288 (1.014–1.636)	0.038
**MALES**							
MetS-free	180	7,805	2.31	1 (reference)		1 (reference)	
MetS-developed	34	1,234	2.76	1.049 (0.734–1.499)	0.792	0.979 (0.685–1.400)	0.908
MetS-recovery	32	889	3.6	1.407 (0.975–2.030)	0.068	1.228 (0.850–1.775)	0.273
MetS-chronic	54	1,436	3.76	1.518 (1.130–2.037)	0.006	1.367 (1.017–1.835)	0.038
**FEMALES**							
MetS-free	203	6,294	3.23	1 (reference)		1 (reference)	
MetS-developed	21	333	6.31	1.835 (1.174–2.866)	0.008	1.527 (0.975–2.390)	0.064
MetS-recovery	10	269	3.72	1.044 (0.555–1.963)	0.895	0.822 (0.436–1.549)	0.543
MetS-chronic	27	419	6.44	1.899 (1.275–2.828)	0.002	1.139 (0.751–1.726)	0.540

*IRR, incidence rate ratio; MetS, metabolic syndrome; TNs, thyroid nodules*.

* *Adjusted for age and sex in all participants. Adjusted for age in the males and females. Age was stratified as 18–30, 31–50 and 51–65 years old*.

In male participants, TN incidence had the same trend. The MetS-chronic group (3.76/100 person-years) showed a higher incidence compared with the MetS-free group (2.31/100 person-years), which was comparable to the MetS-developed and MetS-recovery groups. After adjustment for age, the MetS-free and the MetS-chronic groups still showed a statistically significant difference.

In females, TN incidence rates were significantly higher in the MetS-chronic (6.44/100 person-years) and MetS-developed groups (6.31/100 person-years) compared with the MetS-free (3.23/100 person-years) and MetS-recovery (3.72/100 person-years) groups. After adjustment for age, no significant differences were found among all groups.

### Intergroup Comparisons

In further comparison between study groups with the same initial MetS status (Table 5), the MetS-developed group showed no significant difference in the risk of TNs compared to the MetS-free group. Idem for the MetS-recovery and MetS-chronic groups. These findings indicated that newly developed MetS did not increase the risk of TNs, which was also not reduced by improving metabolic abnormalities.

**Table 5:** TNs risk comparison between study groups.

**Compared subgroups**	**Unadjusted model**		Multivariable model^*^	
	**IRR (95% CI)**	***P***	**Adjusted IRR (95% CI)**	***P***
**Total participants**				
MetS-developed vs MetS-free	1.187 (0.899–1.568)	0.227	0.977 (0.683–1.397)	0.897
MetS-recovery vs MetS-free	1.224 (0.894–1.677)	0.207	1.094 (0.797–1.501)	0.579
MetS-chronic vs MetS-free	1.530 (1.209–1.937)	<0.001	1.288 (1.014–1.636)	0.038
MetS-recovery vs MetS-developed	1.033 (0.691–1.534)	0.875	0.981 (0.656–1.467)	0.927
MetS-recovery vs MetS-chronic	0.840 (0.578–1.219)	0.358	0.865 (0.596–1.257)	0.447
MetS-chronic vs MetS-developed	1.230 (0.873–1.732)	0.236	1.124 (0.796–1.587)	0.506
**MALES**				
MetS-developed vs MetS-free	1.049 (0.734–1.499)	0.792	0.979 (0.685–1.400)	0.908
MetS-recovery vs MetS-free	1.407 (0.975–2.030)	0.068	1.228 (0.850–1.775)	0.273
MetS-chronic vs MetS-free	1.518 (1.130–2.037)	0.006	1.367 (1.017–1.835)	0.038
MetS-recovery vs MetS-developed	1.305 (1.130–2.037)	0.279	1.260 (0.776–2.044)	0.350
MetS-recovery vs MetS-chronic	0.971 (0.627–1.503)	0.893	0.928 (0.599–1.439)	0.740
MetS-chronic vs MetS-developed	1.345 (0.876–2.066)	0.176	1.320 (0.860–2.028)	0.205
**FEMALES**				
MetS-developed vs MetS-free	1.835 (1.174–2.866)	0.008	1.527 (0.975–2.390)	0.064
MetS-recovery vs MetS-free	1.044 (0.555–1.963)	0.895	0.822 (0.436–1.549)	0.543
MetS-chronic vs MetS-free	1.899 (1.275–2.828)	0.002	1.139 (0.751–1.726)	0.540
MetS-recovery vs MetS-developed	0.589 (0.278–1.252)	0.169	0.565 (0.266–1.201)	0.138
MetS-recovery vs MetS-chronic	0.577 (0.279–1.192)	0.137	0.712 (0.338–1.497)	0.370
MetS-chronic vs MetS-developed	1.022 (0.578–1.807)	0.941	0.809 (0.447–1.463)	0.483

*IRR, incidence rate ratio; MetS, metabolic syndrome; TNs, thyroid nodules*.

* *Adjusted for age and sex in all participants. Adjusted for age in males and females. Age was stratified as 18–30, 31–50 and 51–65 years old*.

Comparing study groups with the same MetS status after follow up, the MetS-recovery, and MetS-free groups showed similar risk of TNs (TN risk not increased by MetS), as well as the MetS-developed and MetS-chronic groups (no difference in TN risk between new and long-term MetS).

## Discussion

In agreement with previous studies (16, 17), the cross-sectional part of the present study demonstrated that nodular thyroid disease was more common in MetS patients, and that older age, female gender, and MetS presence were independent factors affecting TN occurrence. In addition, men with chronic MetS have an increased risk of TNs compared with those consistently free from MetS.

The insulin/insulin like growth factor axis, through its mitogenic effect, stimulates the proliferation of thyroid follicular cells. Studies have shown that elevated circulating amounts of insulin promote the formation and growth of TNs (25–27), even in the pediatric population (28). It is likely that the compensatory hyperinsulinemia, which plays a central role in the pathogenesis of MetS (29), is responsible for the rising trend of nodular thyroid disease. The prospective part of the present study found that men with chronic MetS had higher risk of TNs than those consistently without MetS. Besides cardiovascular diseases and type 2 diabetes, an elevated risk of TNs appears to be another complication of chronic MetS status. However, during a median follow up of 5.94 years, no significant increase in the risk of TNs was found for men between newly developed and chronic MetS cases. Recovery from MetS is associated with positive effects on vascular properties (30) and decreases the risk of major adverse cardiovascular events (31). However, we found no significant decrease in the risk of TNs in MetS-recovery men compared to those with chronic MetS. Longer follow up would enable the evaluation of the long-term effects of altered MetS status on the risk of TNs.

It was reported that estradiol promotes the growth of thyroid cells (32–36). Estradiol is considered a potent growth factor both for benign and malignant thyroid cells (37–39). Men with TNs show reduced levels of testosterone and sex hormone-binding globulin (SHBG) (40). The gender disparity observed in the association of chronic-MetS with the risk of TNs may be due to the different impact of MetS on endogenous sex hormones. Indeed, elevated estradiol (41) and decreased testosterone and SHBG (42–45) amounts have been reported in men with MetS. Meanwhile, the levels of testosterone and SHBG are increased in women with MetS (46–48), which may blunt the effect of hyperinsulinemia.

A previous report (15) and the cross-sectional part of the present study suggested MetS as an independent risk factor for TN occurrence in women. However, in the prospective part of the present study, the MetS-developed and MetS-chronic groups in women showed no higher risk of TNs compared with the MetS-free group after adjustment for age. In case the positive effect of hyperinsulinemia on TN formation is blunted by the fluctuation of sex hormones, a median follow up of 5.94 years might not suffice to clearly observe the effects of MetS on TN formation. Therefore, long-term follow up studies, also including younger subjects and children are required to clarify this issue. It should be noted that similar thyroid nodule and autoimmune thyroid disease (AITD) rates were found in polycystic ovary syndrome (PCOS) cases and controls (49).

To the best of our knowledge, this is the first prospective study investigating the association of dynamic MetS status with the risk of TNs. However, this study had several limitations. First, the study population might be biased, as all subjects were individuals undergoing routine health checkup as determined by themselves. Secondly, we could not include 2-h postprandial plasma glucose, which is one of the MetS diagnostic criteria, because of the limited time for administering the oral glucose tolerance test (OGTT) and other medical examinations in one session of health checkup. Thirdly, the sample size was relatively small, with a relatively short follow up. Larger prospective studies with longer follow up would enable the evaluation of the long-term effects of MetS on TNs and thyroid cancers. Fourthly, insulin levels, insulin resistance, and waist to height ratio (WHtR) were not assessed in this study. Therefore, insulin, and thyroid and sex hormones should be examined in further studies.

## Conclusion

The present study showed that nodular thyroid disease is more common in MetS cases, and older age, female gender and MetS were found to be independent risk factors for TN occurrence. In addition, with a median follow up of 5.94 years, men with chronic MetS showed an increased risk of TNs compared with those consistently free from MetS.

## Data Availability Statement

The original contributions presented in the study are included in the article/supplementary material, further inquiries can be directed to the corresponding authors.

## Ethics Statement

The studies involving human participants were reviewed and approved by the Committee on Human Research at Foshan Hospital of Traditional Chinese Medicine. The patients/participants provided their written informed consent to participate in this study.

## Author Contributions

QL, YY, SL, CH, and DH carried out the studies, participated in collecting data, and drafted the manuscript. QL, SY, and SC performed the statistical analysis and participated in its design. LK and DL helped to draft the manuscript. All authors read and approved the final manuscript.

## Conflict of Interest

The authors declare that the research was conducted in the absence of any commercial or financial relationships that could be construed as a potential conflict of interest.
